# Waste-Energy Nexus: Cellulose Wood Chips Conjugated Metal Nanoparticles Based Phase Transformation for Improving Thermal Energy Storage Performance

**DOI:** 10.3390/polym15214291

**Published:** 2023-11-01

**Authors:** Ehssan Ahmed Hassan, Maha A. Tony

**Affiliations:** 1 Department of Biology, College of Science and Humanities, Prince Sattam Bin Abdul Aziz University, Alkharj 11942, Saudi Arabia; 2 Department of Zoology, Faculty of Science, Suez Canal University, Ismailia 41522, Egypt; 3 Basic Engineering Science Department, Faculty of Engineering, Menoufia University, Shebin El-Kom 32511, Egypt; 4 Advanced Materials/Solar Energy and Environmental Sustainability (AMSEES) Laboratory, Faculty of Engineering, Menoufia University, Shebin El-Kom 32511, Egypt

**Keywords:** heating, phase change materials (PCM), thermal energy storage (TES), waste wood chips, nanoparticles

## Abstract

Highlighting waste as a wealth is the future sustainability of the world. Also, using solar energy stored during off-sun periods will overcome the energy crisis. The introduction of wood chip waste for thermal energy storage systems is a sustainable opportunity. Cellulose derived from wood chips was mixed with the environmentally benign magnetite to form a composite (WCM) and mixed with paraffin-based PCM. The composite was characterized through transmission electron microscopy, TEM analysis, scanning electron microscopy, SEM (augmented with dispersive X-ray analysis, EDX). Micrographs, Fourier transform infrared (FTIR), and X-ray diffraction (XRD), which confirmed that the composite material was prepared. Various system proportions of the composite (0.5, 1.0, 2.0, and 4.0%) are embedded in paraffin, and then the thermal system performance is compared. The experimental data revealed that the addition of 2.0 weight percent of composite material showed superior system performance. Also, differential scanning calorimeter (DSC) and TEM analyses of the paraffin-based WCM-composite materials are conducted. The system achieved a heat gain of 87 kJ/min in comparison to 7 kJ/min for 2.0% WCM-PCM and pristine PCM, respectively. Hence, WCM-PCM embedded with waste stream nanoparticles could be suggested as a potential candidate for heating applications.

## 1. Introduction

The superior location of the Middle East countries poses them with the potential for high-renewable energy applications. Such countries are well endowed with sunny weather and a dry climate over the whole year, specifically in the non-coastal regions [1]. Hence, the climate helps in the solar projects especially with the worldwide energy crisis challenges. In this regard, solar energy is a good opportunity to solve the challenges of both environmental pollution control and fossil fuel depletion [2,3,4]. Solar energy as a clean technology with zero emissions is not only a cheap solution, but it could also be widely spread for domestic and industrial distribution [4,5]. Even though a lot of benefits are linked to the solar energy application and uses, the drawbacks, including the high installation cost and inconsistency, are still a deficiency [6,7,8]. Thus, searching for a solution to the non-continuity due to the daytime dependency is a research topic. Thus, such a solution should overcome the gap between energy needs and supply. The idea of storing solar energy might solve those associated problems.

Recently, various energy storage facilities based on phase change materials (PCM) for thermal energy storage have become available. Such substances are capable of storing thermal energy by absorbing or may be releasing the termed “latent heat, LH” [9]. PCM, including organic and inorganic materials such as paraffin and salt hydrates [10,11,12]. Thermal energy storage (TES) across latent heat storage is flexible in application due to their heat recovery with minimal temperature difference through storing and releasing. Hence, such material retains a high energy storage density [11]. Organic PCMs are signified by self-nucleation, which means the crystallization process could occur with or without minimal supercooling [13,14]. Also, organic PCMs have superiority in applications since they are noncorrosive in nature [5,15,16,17]. In this regard, a special concern has been raised for the use of paraffin materials as PCM materials in the TES field due to their reliable thermo-physical properties [18]. Such reliable characteristics include a suitable melting temperature profile with a large amount of LH energy, negligible supercooling, and being stable in room conditions. Also, it is noteworthy to mention that such PCM types have high chemical/thermal performance stability due to their repeatable solidification properties. Paraffin’s PCMs could be classified as paraffin hydrocarbons and paraffin waxes [19]. On the contrary, Paraffin’s PCMs pose demerits, including low thermal conductivity; thereby, the PCM system enhancement is critical to such effectiveness.

Numerous researchers [5,17,18,19,20] have studied the improving paraffin-based PCM system. Congregating the use of nanoparticles for PCM enhancement is a suggested option. Studies are dealing with the enrichment of PCM systems through the inclusion of various types of nanoparticles [20]. The thermal conductivity of nanomaterials plays a vital role in enhancing the conductivity of PCMs. Notably, embedded PCM with nanoparticles not only enhances their thermal conductivity but also modifies various characteristics of the base PCM linked to its thermophysical properties [21,22,23]. The most vital properties include melting/solidification temperatures and phase change periods [24]. Consequently, a controllable dispersion of nanosized particles into the PCM is a suggested opportunity to improve the TES. On the other hand, an undesirable elevation in the dynamic viscosity could be perceived. Moreover, a deficiency in natural convection is attained, which also complicates the melting cycle. Furthermore, the cost of nanoparticles is still gaining scientists’ attention [25,26]. Thus, nano-additives in PCM need more efforts to justify their utilization, which means future research is required to enhance PCM systems.

With the revolution in the material science field and sustainability, the utilization of wood chips as a waste material to be valorized into ultra-value-added material is required [27]. The utilization of wood chips as biomass substances contains holocellulose, cellulose, lignin, and ash [1]. Nanocellulose from wood chips is gaining special attention for its characteristics, including its high surface area, surface charge, and surface energy, especially when augmented with metal-based nanoparticles [28]. Also, Wang and his co-workers fabricated a wood-based material with superior physical and mechanical characteristics [4]. In this concept, introducing nano-cellulose from sawdust-conjugated magnetite nanoparticles in the field of PCM has not been applied so far, according to the authors’ knowledge.

Herein, the current work aims to provide an enhancement in the thermal conductivity of paraffin PCM by adding economic materials from waste streams as a source of nanocellulose and magnetic-based material (magnetite). Such PCM composites are applied to enhance the thermal properties of the solidification and melting cycles, thus enhancing the hot water stored in the system. The composite material is prepared and mixed in different proportions with the base PCM, and the material characteristics are checked through structural and morphological properties. Thereafter, the prepared composite is introduced to enhance the solidification/melting cycles.

## 2. Methodology and Experimental Section

### 2.1. Materials

The waste wood chips are collected from factories for wood processing. Thereafter, the material, after collection, is transferred to the laboratory for processing. Initially, the material is subjected to hydrolysis using hydrochloric acid, followed by bleaching via hydrogen peroxide. Meanwhile, the chemicals ferrous sulfate, ferric sulfate Fe_2_(SO_4_)_3_ (Qualikems Fine Chem Pvt. Ltd., Delhi, India), and NaOH were all supplied as precursors for magnetite preparation. A commercial-grade paraffin wax was used as the base PCM substrate. The material has a melting point of 54 °C and a latent heat (LH) for fusion of 190 kJ/kg.

### 2.2. Synthesis of PCM-Composite

Primarily, wood chips are subjected to successive washings with distilled water to remove any impurities from such waste. Thereafter, the clean wood chips were exposed to overnight electric oven drying before being subjected to chemical treatment. Then, the cellulose fiber is attained through hydrolysis with (2.5 normal) hydrochloric acid and then bleaching with hydrogen peroxide. Adding 200-mL of hydrochloric acid to hydrolyze 25 g of cleaned and dried wood chips, the temperature is raised to 90 °C for 15 min. then, after being filtered, it is subjected to successive washings to reach a pH of 7.0, which is then oven dried. The resultant powder is exposed to bleaching by hydrogen peroxide through heating to 90 °C for 60 min. The mixture after each treatment is washed with distilled water until pH 7.0 is reached. Thereby, the cellulose fibers are dried for use [3,4]. Meanwhile, Fe_3_O_4_ nanoparticles are prepared through the co-precipitation technique. 2 mol of ferrous and 1 mol of ferric sulphate at their stoichiometric ratios were dissolved in distilled water. The mixture is subjected to stirring under heating with the drop-wise addition of NaOH into the mixture solution till pH 11.0 is attained and subjected to continuous stirring at 80° to attain a precipitate. The precipitate is formed, which is collected and washed successively with distilled water until pH 7.0 is reached prior to drying to attain the fine powder of Fe_3_O_4_ nanoparticles [5]. To attain the composite, cellulose wood chips/magnetite (WCM), physical mixing to attain a homogenous blend was followed by hydrothermal mixing of two to one of the woods using mixing chips to one magnetite weight percent. Subsequently, a droplet of water is added to the mixture, which is then heated in a microwave oven for 5 min at 500 W.

**Figure 1:**
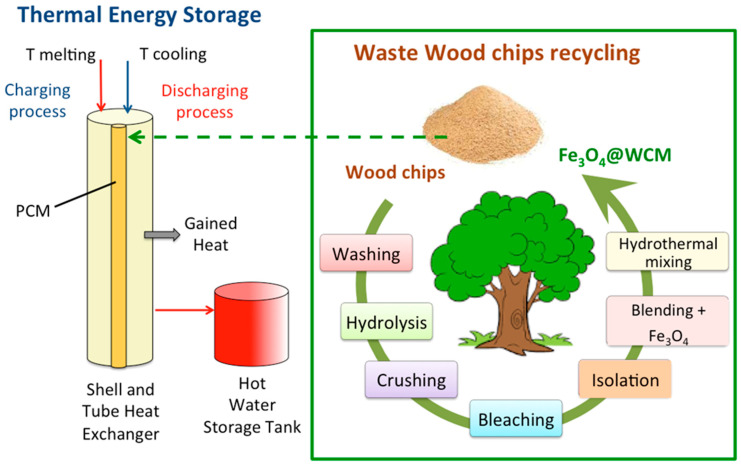
Schematic representation of the procedure of magnetite/wood chip composite PCM opportunity.

The solid mixture sample of WCM is added to the paraffin PCM to form a composite PCM mixture labeled PCM-WCM. The PCM-WCM was prepared at different WCM proportions ranging from 0.5, 1.0, 2.0, and 4.0 weight %. Then, the mixture is sonically mixed at 60 °C under ultrasonic radiation (WUC-A03H, 40 kHz Bernolsheim, France). The schematic steps of the PCM preparation and system are illustrated graphically in Figure 1.

### 2.3. Characterization

X-ray powder diffractometry XRPhillips X’pert (Cambridge, UK) (MPD3040) diffractometer with Cu-K_α_ radiation at room temperature (λ = 1.5406 Å) and in step-scan mode was used to investigate the structure of the samples. Field-emission scanning electron microscope (SEM), (FE-SEM, Quanta FEG 250, Beijing, China) and transmission electron microscopy TEM (type Tecnai G20, FEI) are used to investigate the morphologies of pristine wood chips and magnetite nanoparticles or composite material WCM. Also, Fourier transform infrared (FTIR) spectra (Jasco, FT/IR-4100, type A, Beijing, China) exhibited the type of functional groups of the WCM.

## 3. Results and Discussion

### 3.1. Characterization of WCM Substance

#### Structural and Morphological WCM Characterization

**Structural characterization using XRD pattern:**

**Figure 2:**
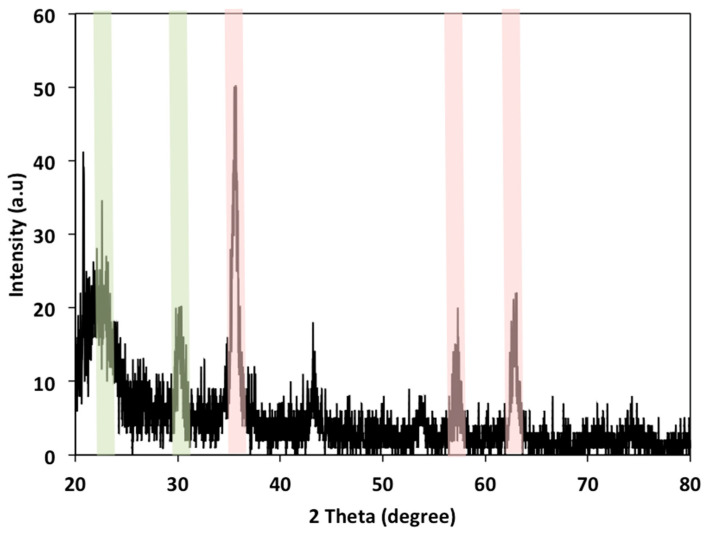
X-ray diffraction patterns of magnetite/wood chips composite for PCM.

Figure 2 displays the XRD pattern for composite cellulose wood chips conjugate magnetite nanoparticles. As displayed in the pattern, the 2θ values of 34.7° and 22.4° reveal the presence of cellulose material [1]. Also, it is obvious from the graph of XRD that the spinel structure of ferrite exposed as a single face-cantered cubic (fcc) is estimated. The presence of the intensive peaks at 2θ of 35.52, 62.84, and 30.2° corresponds to 311, 440, and 220, respectively, as well as the presence of 54.5, 66.7, and 74.8°, which represent the crystal plans of 422, 511, and 533, 2. Such plans perfectly match the standard magnetite peaks that confirm the presence of magnetite in the sample.

**Morphological characterization using SEM micrographs:**

**Figure 3:**
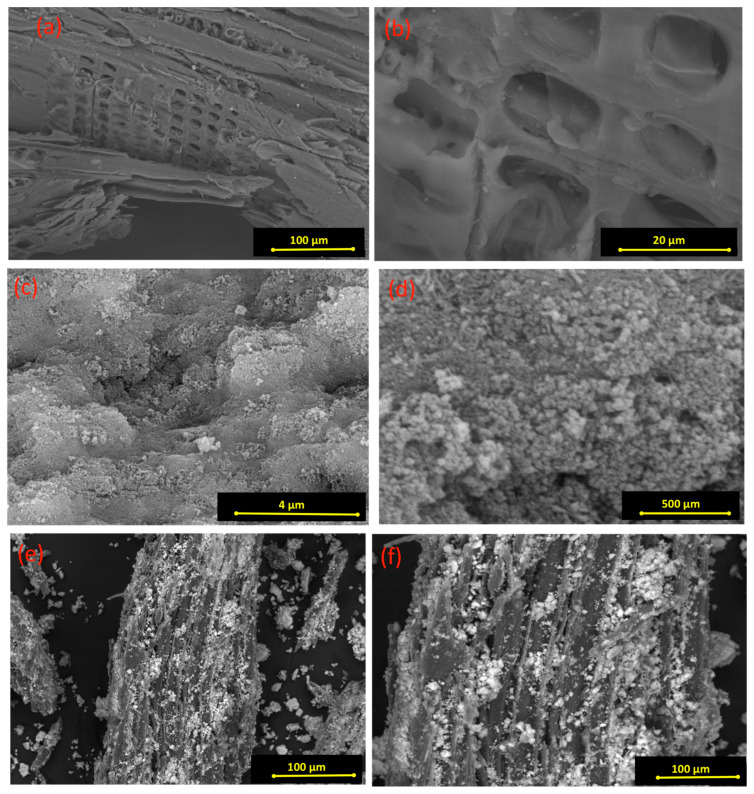
SEM images of magnetite/cellulose wood chips composite for PCM (**a**,**b**) pure wood chips; (**c**,**d**) pure magnetite; and (**e**,**f**) composite WCM.

SEM micrographs were retained at different magnifications for the pure wood chips, the pure magnetite, and the composite material. Figure 3 displays the SEM images to illustrate the surface morphology of both pristine and conjugated composite material. According to the data displayed in Figure 3a,b, the pristine wood chip material consists of layers, and between such layers are numerous arranged vessels. However, Figure 3c,d, the magnetite nanoparticles consist of sphere-like particles. Also, it is significant to mention that the vessel and shape of the pure wood chip material are smooth, with loose folds between such vessels. As illustrated in Figure 3e,f, the micrographs of the cellulose wooden chips augmented with the magnetite nanoparticles (WCM) show that a gloss change of the surface was attained in the wood chips due to the combinations of the magnetite with the vessels of the wood chips, which means a change in the intrinsic structure of the composite rather than in the pristine cellulose wood chips. Such micrographs verify the fruitful mixture of wood chips and magnetite nanoparticles.

**Morphological characterization using TEM micrographs:**

**Figure 4:**
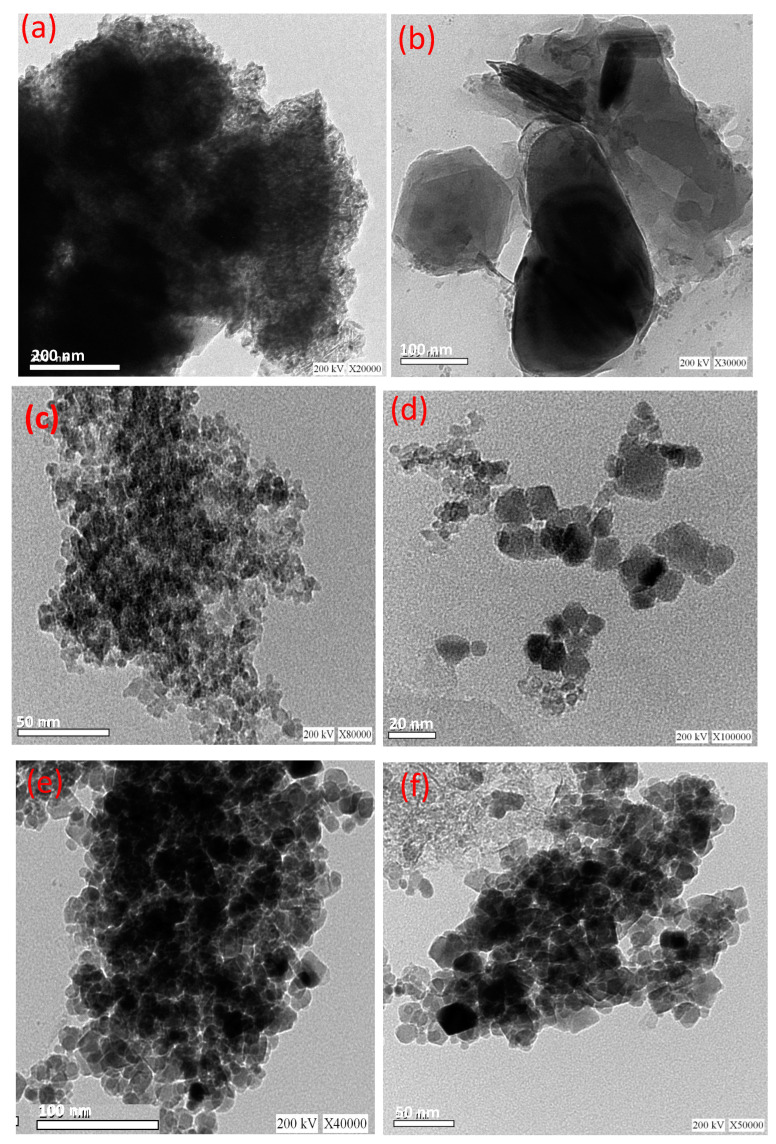
TEM images of magnetite/cellulose wood chips composite for PCM (**a**,**b**) pure wood chips; (**c**,**d**) pure magnetite; and (**e**,**f**) composite WCM.

Figure 4 displays the TEM micrographs at different magnifications for the pure cellulose-based wood chips, pure magnetite, and the composite material. Figure 4a,b display the micrographs of wood chip material at various magnifications. The micrographs in Figure 4a,b display a sheet-like material. Figure 4c,d exhibited the TEM micrographs of magnetite nanoparticles at different magnifications. The images display a uniform, spherical-like shape for the nanoparticles. It is obvious from the images in Figure 4e,f of the hybrid composite that the spherical-like shape of magnetite nanoparticles is distributed in the wood chip substance fiber. Such images verify the sheet-like morphology of the wood chips decorated with magnetite nanoparticles in spherical shape.

**Fourier transforms infrared measurements**

**Figure 5:**
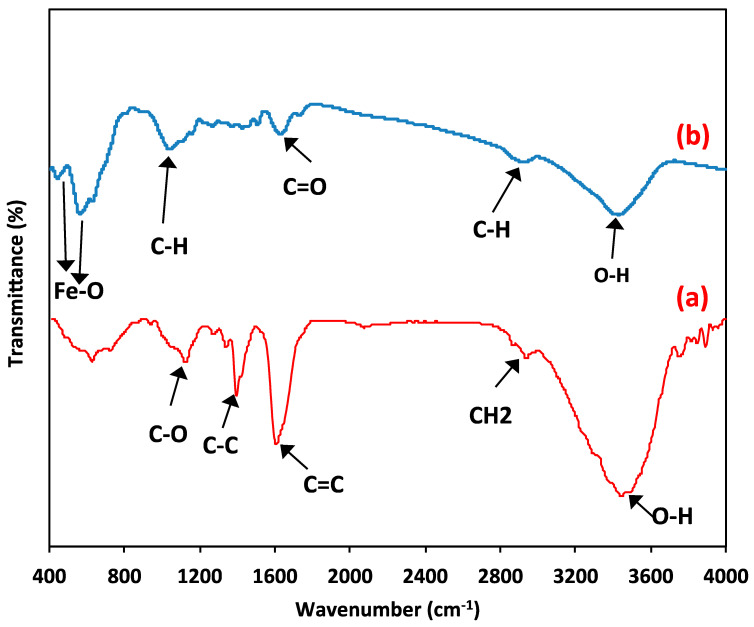
FTIR spectrum of (**a**) wood chips; (**b**) magnetite/wood chips composite for PCM.

In order to categorize the functional groups of the prepared composite material, FTIR spectra (Figure 5) were conducted. Firstly, the FTIR spectra of the raw wood chip material are investigated, and the data are displayed in Figure 5a. The results revealed a broad band at 3433 cm^−1^, which suggests the presence of OH stretching of the cellulose and lignin groups. Also, the absorption band at 2924 cm^−1^ signifies the stretching of the aliphatic CH2 group. However, the bands at 1612 cm^−1^ and 1509 cm^−1^ due to the C=C stretching in the phenol group and the C=C stretching in the aromatic compounds. Further bands at 1735 cm^−1^ are due to the stretching of the aldehyde C=O group. Furthermore, the presence of a band of the C-O group at 1041 cm^−1^ signifies the carboxylic acids and alcohols present in natural wood chips.

Additionally, for the composite material (wood chips conjugated with magnetite), the FTIR spectrum is presented in Figure 5b. Cellulose is the predominant substance in wood chips. Further, in the composite magnetite-conjugated cellulose. Thus, the FTIR graph exhibited O-H symmetry, aliphatic C-H stretching, and C=O vibration that corresponded to the wavenumber numbers of 3370 cm^−1^, 2906.2 cm^−1^, and 1732.73 cm^−1^, respectively. The peaks of 431.97 and 555.39 cm^−1^ verify the presence of magnetite nanoparticles and reflect magnetite presence [1].

### 3.2. Thermal Energy Storage (TES) Performance

#### 3.2.1. Melting/Solidification Cycles

**Figure 6:**
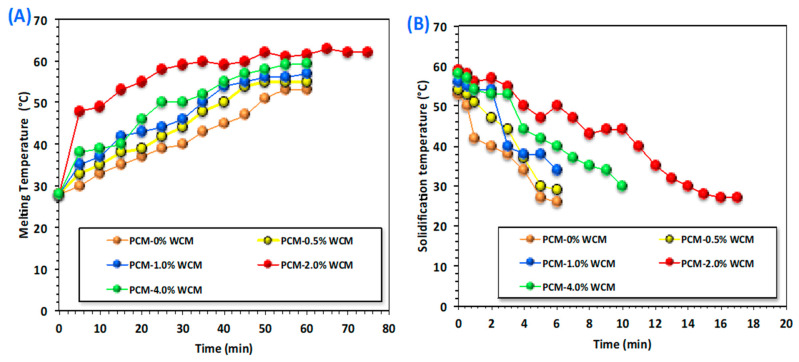
Melting (**A**) and solidification (**B**) cycles for charging and discharging of pristine and embedded nanoparticles of PCM.

Energy storage and the rate of heat release of the phase transformation materials are linked to the melting and solidification cycles, depending on the temperature difference between the heat transfer fluid and the phase change material as well as their thermal conductivities [29,30,31,32,33,34]. Thus, in this concept, the melting and solidification temperatures for the charging and discharging cycles of the temperature variation of the PCM are assessed, and the results are displayed in Figure 6A,B. The data investigated for the PCM composite (PCM-WCM) with respect to the time period of melting and solidification at different times were compared to that for the pristine paraffin wax PCM. Wood chip conjugates of magnetite at different proportions (0.5, 1.0, 2.0, and 4.0 weight %) are embedded into the base PCM paraffin wax. Notably, it is observed in Figure 6A. Wood chip conjugates magnetite nanoparticles in different proportions, resulting in a significant range of melting temperatures. An elevation in the temperature was noted by the WCM addition to paraffin-based PCM. The most pronounced system was noted for the 2.0-weight % addition of WCM-augmented PCM. The charging temperature was elevated to 63 °C, compared to 53 °C for the pristine PCM. However, the increase reached only 55, 57, and 59 °C when the embedded nanofiller of WCM in the PCM was 0.5, 1.0, and 4.0 weight %, respectively. This means that above 2.0-weight % addition to the PCM, the melting temperature starts to diminish. Particularly, nanoparticle addition to the base paraffin PCM helps ensure a modification in the shape of the heat flow of the PCM [35,36,37]. Therefore, the addition of nanoparticles adapts the value of the PCM’s melting temperature. Additionally, it is estimated that the nanoparticles inserted in the base PCM improve its latent heat and control its photodegradation [38,39], which will be more effective in real-world applications when solar energy is the source of the melting cycle.

Figure 6B displays the data on the discharging cycle of both pristine paraffin and composite paraffin PCM. It is clear from the results of the experimental discharging cycle that there is an increase in the melting temperature of the PCM with the addition of WCM nanoparticles. Thereby, the solidification temperature will be further enhanced compared to that of pure PCM. However, different temperatures were observed with the different weight percent of the embedded nanoparticles. Hence, the process is dependent on the weighted % addition of the nanoparticles to the PCM material. Accordingly, the overall heat stored by the system is enlarged, with a significant improvement that corresponds to a 2.0% cellulose-WCM addition to the paraffin. However, an extra increase in the added nanoparticles results in a decline in the system, and the process becomes unfavorable. Such an investigation was previously elucidated by scattered authors [5,40,41,42] since the extra nanoparticles might reduce the stability of the phase change material. This could be attributed to the excess embedded nanoparticle filler, which results in agglomeration and sedimentation in the paraffin PCM material. Hence, selecting the optimum value of additive nanoparticles in PCM is required for enhancing the melting and solidification cycles that correspond to improving the charging and discharging processes, respectively. This result is in accordance with what was previously reported in the literature [31,43,44,45,46]. It is noteworthy to declare that extra nanoparticles in the base of PCM might be leading to an elevation in the dynamic viscosity of the phase change material. Thereby, such an increase in the dynamic viscosity could deteriorate the heat transfer rate of the phase change material.

**Figure 7:**
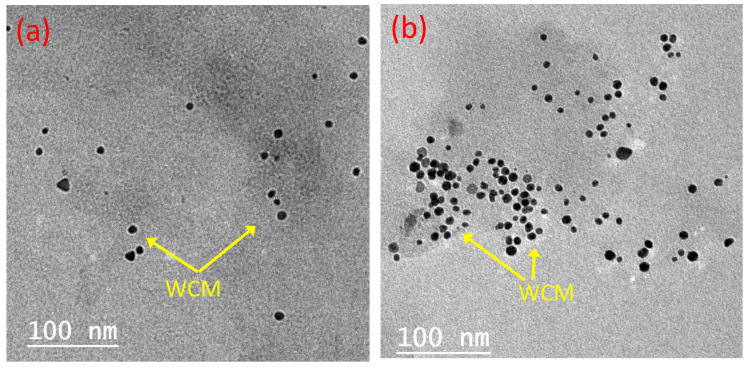
TEM images of PCM composite conjugated with different magnetite/cellulose wood chips nanoparticles (**a**) 2.0% magnetite/cellulose wood chips nanoparticles; (**b**) 4.0% magnetite/cellulose wood chips nanoparticles.

Transmission electron microscope (TEM) analysis for the PCM compromises of 2.0% WCM and 4.0% WCM was conducted to conclude and confirm the optimized formulation. The images of the two composite PCM types are displayed in Figure 7. According to Figure 7a,b, the TEM images showed that according to the dispersed percent of the cellulose-WCM in the paraffin PCM (2.0 or 4.0%, respectively), the nanoparticles are well dispersed in a scattered manner in the PCM up to 2.0%. However, excess particles in the base paraffin PCM result in the accumulation and agglomeration of the nanoparticles. Thus, the thermal system efficiency has declined. Therefore, the improvement in the base PCM corresponds to a 2.0% cellulose-WCM addition to the paraffin. In this concept, TEM analysis verified the agglomeration of the WCM on the base PCM (paraffin wax) when the mass percent was greater than 2.0%.

**Figure 8:**
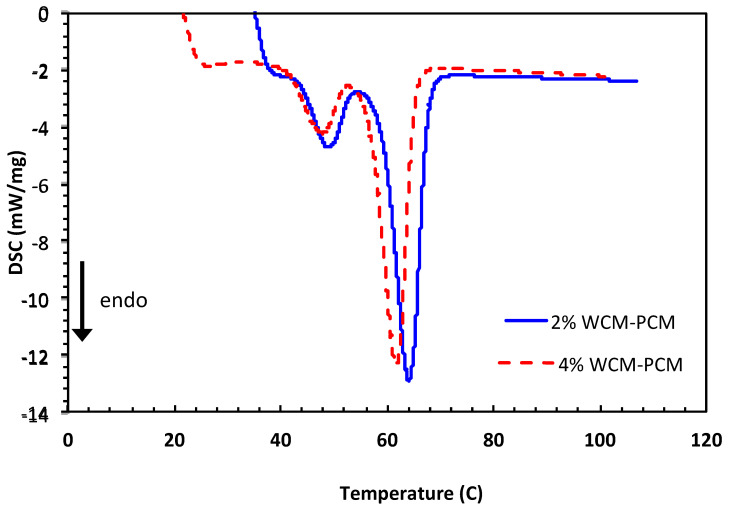
DSC curves of PCM composite conjugated with different magnetite/cellulose wood chips nanoparticles 2.0% and 4.0% magnetite/cellulose wood chips nanoparticles.

In order to further illustrate the role of the cellulosic magnetite proportion in the PCM-based paraffin’s, differential scanning calorimeter (DSC) measurements are conducted at a constant heat rate corresponding to 10 °C/min. DSC measurements were carried out on both PCM composites dispersed at 2.0 and 4.0% to attain data that verifies the results of the melting and freezing temperatures of the WCM-PCM material. The DSC data displayed in Figure 8 show that each DSC curve has two endothermic peaks. Such two peaks are signified by the solid–solid transition, which is then followed by the solid–liquid transition cycle. In general, solid–solid transition happens by changing temperature, which is a polymorphic feature for the material because of phase transformation from a certain crystalline solid configuration into another form of crystalline solid. Such crystalline solid–solid transitions are associated with erratic variations in volume, enthalpy, and entropy as a result of crystal packing modifications. However, it is noteworthy to mention that those variations possess minimal values in comparison to the corresponding solid–liquid transition cycles.

According to the data shown in Figure 8, peak and endset temperatures shifted to higher temperatures. When comparing 2% WCM-PCM to 4% WCM-PCM, it is observed that the phase change temperature of the 2% WCM-PCM-based phase transition composite is 61.4 °C and increases to 60.13 °C when 2% WCM-PCM and 4% WCM-PCM are added, respectively. Based on the typical DSC thermogram data, the results confirm the agglomeration of the WCM, with the optimum value (2%) dispersed in the base PCM. Thus, above the optimum value of loading, the melting process becomes fast, which hence reduces the storage capacity.

#### 3.2.2. Heat Profile Yield

**Figure 9:**
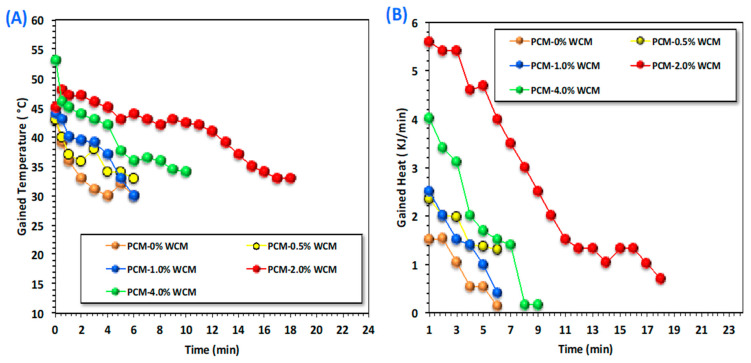
Heat storage diagrams through (**A**) temperature gain and (**B**) heat flow rate for pristine and embedded nanoparticles of PCM.

Figure 9 explores simultaneously the gained temperature and the sum of heat rates released by the phase change material through the discharging cycle so as to deduce and verify the optimal performance for real applications. The data displayed in Figure 9A,B is for temperature gain and heat released, respectively. Accordingly, for choosing the optimized value of nanosized additives based on cellulose-wood chips augmented magnetite nanoparticles, it might be reflected through heat performance and the range of phase transformation temperature gained and phase transformation heat. The experimental data expose that nanoparticle addition to the paraffin-based PCM substance can enhance both the range of temperatures and the amount of heat gained by the system. Thereby, such material can be applied for heat storage through a hot water storage facility. Moreover, the embedded nanofiller increased the temperature, which means an enhancement and elevation in the stored heat in comparison to the pristine PCM (Figure 9A).

For maintaining the optimum heat storage capacity, as illustrated in Figure 9B, the heat gained from the PCM is increased from 1.33 to 1.03 kJ/min for pristine paraffin and 2.0% WCM-augmented paraffin’s PCM. Such a result might be attributed to the enhancement, which confirms a higher thermal transfer rate that is elevated with the amount of embedded nanoparticle material in the PCM, especially in the optimal weight percent [47]. Hence, the addition of WCM nanoparticles to pristine paraffin wax to enhance the heat storage performance has notable effectiveness on the stored hot water for further heating in real life. Furthermore, the difference in both temperature and heat gained in the various studied systems is due to the amount of nano-capsules embedded in PCM, and the maximum capacity attained according to the above-mentioned is linked to the optimal 2.0 weight percent filler [48]. This is due to the synergistic effect of the composite substance in the base PCM material [31,49].

### 3.3. Overall Heat

**Figure 10:**
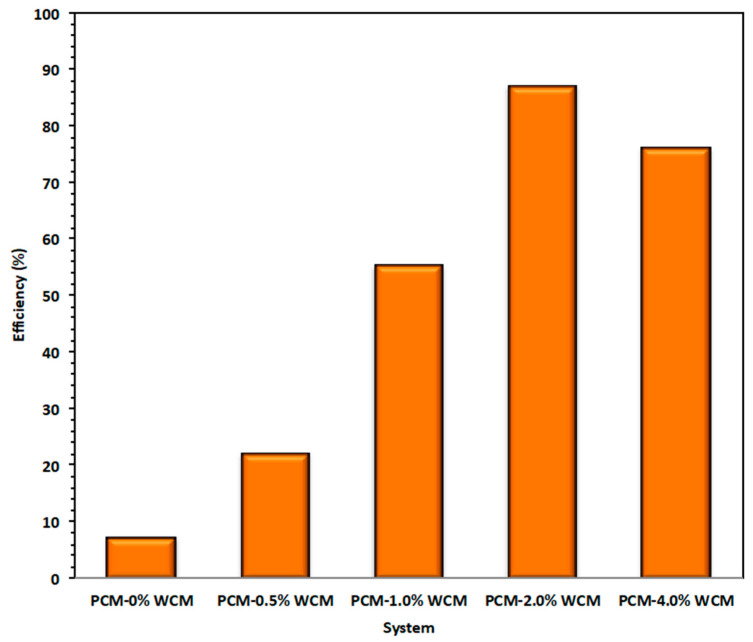
Various heat gained by the PCM based systems.

Overall heat gained from the process is compared for all the studied PCM systems, and the results are displayed in Figure 10. The data illustrated in Figure 8 reveal that the heat transferred by the working fluid (water) that is calculated for the whole process is attained by Equation (1) [50].

(1) $$Q\upsilon =\dot{m}\;Cw\theta_{w}$$

where: $\dot{m}$: mass flow rate of working fluid (g/s); *θ*: inlet and outlet water temperature difference of inlet and outlet heat exchanger; and Cw: Specific heat capacity of water as the working fluid (4.18 kJ/kg·K).

**Figure 11:**
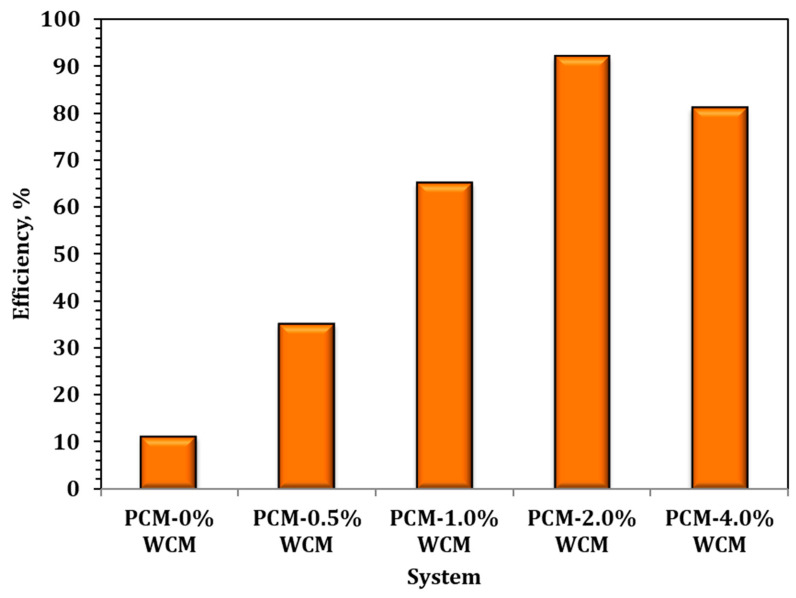
Overall system efficiency for various PCM types.

The overall heat gain phase change material is the heat assigned by the heat transfer fluid and is determined for the whole process by Equation (2). The pristine paraffin and paraffin-augmented nanoparticles in WCM, which showed nano-additive properties to the paraffin, improve the overall heat rate gained from the PCM system. According to the experimental data, the useful rate of heat gain is greater for composite WCM-PCM with a percent addition of 2.0 wt %, which corresponds to 87 kJ/min in comparison to 7 kJ/min for the pristine paraffin PCM system. This heat rate is enhanced proportionately with the embedded weight of the nanoparticles. This investigation is in accordance with what has been previously reported in the literature [14].

(2) $$Q_{PCM}=mC_{p}\left(T_{PCMi}-T_{PCMo}\right)+m\;H$$

where m is the thermal phase change mass (Kg), C*p* is the specific heat capacity of PCM (kJ/kg·K), *T_PCMi_* and *T_PCMo_* are temperatures of the phase change material of the inlet and leaving heat exchanger, respectively, and *H* is the latent heat of fusion of PCM (kJ/kg). Then, the overall storing efficiency (¥) of nanoparticles embedded-PCM system over the solo paraffin PCM is achieved according to Equation (3) [50]. Hence, ¥ is corresponding to the nanofiller of 2.0%-PCM as illustrated in Figure 11.

(3) $$¥=\frac{Q_{\upsilon }}{Q_{PCM}}\times 100$$

For the purpose of verifying the current system performance, the studied PCM systems that have been previously published in the literature are compared with the currently introduced PCM type. According to the cited data, various organic materials are introduced as the organic, inorganic, and eutectic mixture-based PCM type, such as paraffin’s, ƞ-octadecane, petroleum wax, polyurethane, capric acid, and others that are used as the base PCM. Also, various additions are presented to improve the PCM efficiency, including graphite, carbon nano fiber, Al_2_O_3_, CuO, ZnO, and various nanoparticles. The results of the above-mentioned data are tabulated in Table 1. The data presented in Table 1 illustrate that while some other systems showed a superior enhancement than the suggested current work, a considered reasonable improvement is suggested from the work since it is an industrial ecology technique based on using waste material. Thus, the proficient current system has the greatest value since it converts waste into value-added material.

## 4. Conclusions

A sustainable waste-to-energy nexus approach has been investigated and introduced in the current investigation. The study is based on using wood chip waste and converting it to a composite based on cellulose wood chip magnetite material (WCM) as a filler for the PCM. A composite-based paraffin embedded with a different weight proportion of WCM (0.5, 1.0, 2.0, and 4.0%) was prepared for various PCM systems. The superior efficiency is explored, and the maximum thermal behavior displayed is signified as corresponding to 2.0% WCM-PCM system. The proposed system showed a pronounced efficiency, and the maximum performance was 87 kJ/min. Thus, such a system introduces waste-to-wealth criteria to attain a sustainable environment.

## Figures and Tables

**Table 1:** Comparative investigation of various PCM systems compared with the suggested current work with the introduced applications.

PCM		Additives		Application	General Comments	Ref.
Name	Type	Type	Amount (%)			
Paraffin wax	Organic	Waste Cellulose chips/magnetite	2%	Heating	Enhancement in overall heat stored	current work
BaCl_2_	Inorganic	MgO	1%	Supercooling	Enhancement in thermal conductivity	[10]
n-octadecane paraffin	Organic	Al_2_O_3_	2%	Heating	Enhancement in thermal conductivity	[51]
Mn(NO_3_)_2_	Inorganic	Fe_3_O_4_	1%	Cooling	Decrease in temperature	[52]
Polyurethane	Organic	Graphite	1%	Heating	Enhancement in thermal stability	[53]
Paraffin wax	Organic	ZSM12	Na	Heating	Higher solidification temperature	[38]
Lauric & stearic acid	Eutectic	CuO	1%	Heating	Enhancement thermal conductivity	[32]
Capric Acid	Organic	CuO	Na	Cooling	Reduction in temperature of the material surface	[53]
Paraffin Wax	Organic	Carbon nanotubes	2%	Heating	Increase in storage capacity	[54]
Water	Organic	Graphene oxide	Na	Supercooling	Reduction in melting temperature	[55]
Capric acid & oleic acid	Eutectic	Activated carbon nanosheets	0.1%	Sub-cooling	Enhancement in thermal conductivity	[56]
CaCl_2_	Inorganic	γ-Al_2_O_3_	1%	Reductions in latent heat	Supercooling	[10]
Petroleum wax	Organic	α-Al_2_O_3_	2%	Heating	Enhancement in thermal conductivity	[57]
Paraffin wax	Organic	Al_2_O_3_	1.0%	Na	Reduction in melting temperature	[19]
Paraffin wax	Organic	ZnO	2.0%	Na	Higher solidification temperature	[19]
Poly-α-olefin	Organic	Indium	30%	Na	Double in heat transfer	[58]
Ethanol	Organic	Ag nanowires	62%	Heating	Enhancement in thermal conductivity	[59]
Paraffin-tailing ceramic	Organic	nano-graphene	Na	Water heating	Ehnacmeent laternt heat and thermal conductivity	[59]

Na: Not available.

## Data Availability

Data are available upon request.
